# Dual-Energy CT: What the Neuroradiologist Should Know

**DOI:** 10.1007/s40134-015-0097-9

**Published:** 2015-03-18

**Authors:** Alida A. Postma, Marco Das, Annika A. R. Stadler, Joachim E. Wildberger

**Affiliations:** Department of Radiology, Maastricht University Medical Centre, PO Box 5800, 6202 AZ Maastricht, The Netherlands

**Keywords:** Dual-energy CT, Virtual non-contrast, Iodine overlay, Circle of Willis, Bone removal, Intra-arterial recanalization

## Abstract

Because of the different attenuations of tissues at different energy levels, dual-energy CT offers tissue differentiation and characterization, reduction of artifacts, and remodeling of contrast-to-noise ratio (CNR) and signal-to-noise ratio (SNR), hereby creating new opportunities and insights in CT imaging. The applications for dual-energy imaging in neuroradiology are various and still expanding. Automated bone removal is used in CT angiography and CT venography of the intracranial vessels. Monoenergetic reconstructions can be used in patients with or without metal implants in the brain and spine to reduce artifacts, improve CNR and SNR, or to improve iodine conspicuity. Differentiation of iodine and hemorrhage is used in high-density lesions, after intra-arterial recanalization in stroke patients or after administration of contrast media. Detection of underlying (vascular and non-vascular) pathology and spot sign can be used in patients presenting with (acute) intracranial hemorrhage.

## Introduction

With the introduction of dual-energy CT (DECT) in neuroradiology, imaging of various important indications shifted back from MRI to CT and new indications for CT scanning were emerged.

For neuroradiologists in today’s practice, MRI is the cornerstone of imaging. It allows direct imaging of the entire brain and its feeding vessels, and grants for functional imaging as perfusion, diffusion, and spectroscopy. Its ability in tissue characterization in the brain is one of the reasons MRI has replaced CT in clinical routine imaging for most indications. However, CT remains the preferred imaging method in the acute setting, e.g., in neurotrauma, in patients presenting with signs of acute hemorrhage and in patients with contra-indications to MR imaging. However, with the introduction of DECT, tissue characterization became feasible and CT indications are again adapting.

Neuroimaging before CT was limited to indirect imaging of the neuraxis. Diagnostic possibilities mostly consisted of conventional X-rays, myelography, pneumoencephalography and arteriography.

Even after the introduction of the first CT-scanner in 1971, due to the long acquisition time, only imaging of the brain was possible. In 1974, technical progress allowed whole-body imaging and in 1975, for the first time, syringomyelia was detected on CT [1, 2].

In the next decades, CT underwent substantial technical improvements, with its most important advancements: helical acquisition, which became feasible due to the implementation of the slip-ring technology and furthermore the introduction of multidetector-row CT (MDCT). In general, MDCT has improved spatial and temporal resolution as well as z-coverage. Isotropic voxels allow multiplanar reformations (MPR) and 3D imaging. Perfusion of the brain and dynamic (“4D”) imaging of the brain are nowadays commercially available and increasingly used in daily routine. Although the concept of dual-energy (DE) scanning was already described in the late 1970s, due to lack of computational power, broad clinical implementation was not possible until the early 2000s [3–6].

CT scanners with DECT, based on the use of two simultaneous working X-ray tubes (Siemens Healthcare), fast peak kilovoltage switching (GE Healthcare, Toshiba), as well as dual-layer detector systems (Phillips Healthcare) were introduced.

*The advantage of DECT is the ability for material characterization and differentiation, based on high*- *and low*-*peak voltage acquisitions. Materials with equal Hounsfield densities at 120* *kVp imaging can be differentiated by analyzing energy dependent changes of the attenuation of materials (e.g. 80 vs 140* *kVp).*

The neuroradiological indications for DECT imaging lag behind other indications as abdominal imaging, although the use of DECT in neuroradiological and head neck imaging is increasingly valued and literature is expanding [7–12, 13•, 14, 15•, 16••, 17, 18].

On conventional CT hemorrhage and iodine are difficult to discriminate due to similar Hounsfield densities. DECT grants characterization and differentiation of materials, like iodine, calcium and hemorrhage [19–21].

Applications allows bone removal in CT angiography (CTA) and CT venography (CTV) [22–30]. Iodine can be virtually extracted from the images, resulting in iodine maps (IOM). These can be subtracted from the blended images (synonym: mixed/combined images), resulting in virtual non-enhanced images (synonym: virtual non-contrast, VNC). These images discriminate hemorrhage from iodine after intra-arterial recanalization (IAR) in stroke patients [13•, 15•]. The iodine images are useful in patients presenting with intracranial hemorrhage (ICH) to improve the detection of underlying (vascular and non-vascular) pathology and the spot sign, a sign of active bleeding [16••, 31]. Calculation of monoenergetic images can be helpful in CTA because of increased vascular opacification at lower energies [14, 32]. In patients with metal implants, the use of higher kV at monoenergetic imaging increases the delineation of the metal implants and reduces beam-hardening artifacts [33–35], e.g., in CT myelography in patients with metal spondylodesis [12].

## Technical Background

Conventional MDCT uses a polychromatic X-ray spectrum, provided by one X-ray tube. DECT uses two different energy spectra, produced by two different kVp settings either by two X-ray sources or by other techniques, as mentioned below. Typical energies used are 80–100 kVp for lower energy spectrum and about 140–150 kVp for the high-energy spectrum. The attenuation of tissues is energy dependent; at the higher energy spectrum, the attenuation of tissues is lower, whereas attenuation is increased at the lower energy spectrum.

DECT can be accomplished with several different techniques, vendor dependent.

Using two almost perpendicular X-ray tubes provides simultaneously two energy spectra with high and low kVp (e.g. 140 and 80 kVp) (Siemens Healthcare). In a one-tube system, DE scanning can be accomplished by fast kV switching, in which the energy of the X-ray tube changes between 80 and 140 kVp in a near-real-time mode (<0.5 ms) (Toshiba, GE Healthcare). In a dual-layer detector system, the detector consists of two scintillation layers, one layer is capable of measuring the intensity of the low-energy photons and the other of the high-energy photons, to acquire DE data from a single polychromatic X-ray source. Another, non- ‘real-time’-option is single source double scan or sequential scanning [36, 37].

The attenuation of materials is based on the Compton scattering and the photoelectric effect. The photoelectric effect, responsible for the largest part of the attenuation, depends on the energy of the X-ray beam and the atomic number (*Z*) of the tissue and materials. The Compton effect prevails in materials with lower atomic number. In materials with higher atomic number, the attenuation of the X-ray beam is larger, mainly because of the photoelectric effect. When the photon energy exceeds the binding energy of the K-shell electron of the material, attenuation suddenly increases. This effect is called the K-edge.

**Fig. 1:**
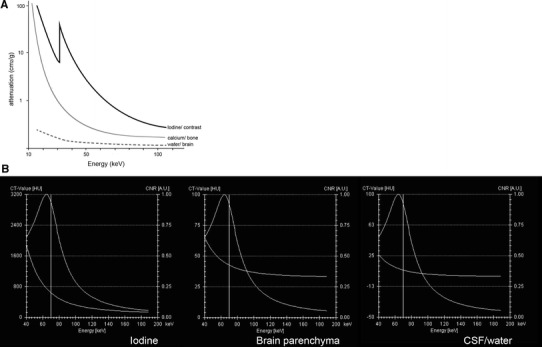
**a** Graph showing the attenuation coefficient of iodine, calcium, and water plotted against energy. The K-edge of iodine is at 33.2 keV, of calcium at 4 keV (not on graph). At lower keV, especially reaching its K-edge, the attenuation of iodine is increasing as opposed to calcium and water. The attenuation coefficients at 40 and 60 keV are for iodine significantly higher than for calcium, providing the possibility for material differentiation in DECT. **b** Graphs represent Hounsfield densities (*left bar*) of iodine (*a*), brain parenchyma (*b*), and CSF (*c*) from same patient as Fig. 8 at different monoenergetic energies. The *right bar* depicts the CNR. Iodine has the highest densities at low monoenergetic energies, as is expected from **a**. CNR decreases at higher energies and is identical in iodine, parenchyma and CSF

DECT uses the variability of the K-edge of different substances and therefore the difference in attenuation of the beam (Fig. 1) [38]. The K-edge of iodine (33.2 keV, *Z* = 53) and calcium (4 keV, *Z* = 20) are sufficiently different from the K-edge of the soft tissue (0.01–0.53 keV) to allow for discrimination on DE imaging.

In material quantification, the concentration of the elements can be determined and quantified.

Iodine has a high atomic number, whereas brain parenchyma, brain hemorrhage, and CSF have low atomic numbers [39]. When two materials with low atomic numbers with different attenuations at a certain energy are put in a diagram with a material with high atomic number (calcium or iodine), the algorithm can decompose a single voxel in a mixture of these three elements and the contribution of the high-density material (calcium or iodine) can be calculated [17, 21], this being called three-material decomposition. The density of the voxel and the amount of the high atomic number substance can be reconstructed. Thus, it is possible to calculate a blended image, a calcium or iodine map. By subtracting calcium or iodine from the blended image, a virtual non-calcium or virtual non-enhanced image (VNC) is generated.

In virtual monoenergetic or monochromatic imaging, images from a DECT dataset are reconstructed at different virtual monoenergies. These monoenergetic images reflect the HU values of the scanned region at one specific energy, opposed to the HU values derived at a polychromatic spectrum. Calculation of these images is based on material decomposition and the specific mass attenuation of these materials, respectively [40].

Recently, an advanced method for calculation of monoenergetic images was published, in which the advantages of high CNR at low keV are combined with the high SNR at higher keV using a frequency split principle [41]. The low keV and the high keV images are decomposed into two sets of subimages. The first set contains only lower spatial frequencies and thus object information, the second set contains the remaining high spatial frequencies, image noise. By combining the lower frequency range from the low kV with the higher frequency range of the higher kV, the benefits of both result in better CNR at higher SNR [41].

Monochromatic images have the potential to facilitate quantitative measurements and reduce beam hardening artifacts, in particular artifacts from metallic structures [40].

## Clinical Applications

### Material Characterization and Differentiation

Material characterization and differentiation is one of the most important advances of DECT compared to standard MDCT in neuroradiology and is used in various indications.

#### Bone Removal CTA

The numbers of CTA over the past decade increased, and CTA of the intracranial and extracranial vessels largely replaced digital subtraction angiography (DSA) for diagnostics.

There are various ways of looking at CTA images: axial source images, MPR, maximum intensity projections (MIP), and volume rendering (VR). Especially in MIP and VR, the bone can hamper interpretation of the vessels. Previously, various methods have been used to remove the bone of skull and skull base to facilitate interpretation of the vessels [22, 42, 43].

Commonly applied methods of bone removal are

Manual segmentation of bone and vessels. This method is time consuming and operator dependent.

Semiautomatic segmentation by (vendor specific) threshold-based software based on density differences of structures. Disadvantages are possible subtraction artifacts when HU of vessels, calcified plaques and bone are very similar.

Subtraction segmentation, subtracting the non-enhanced CT (NECT) from the contrast enhanced CT (CECT). Even though this method is not threshold dependent, radiation dose is increased and vulnerable for motion artifacts between the two scans [22, 42, 43].

**Fig. 2:**
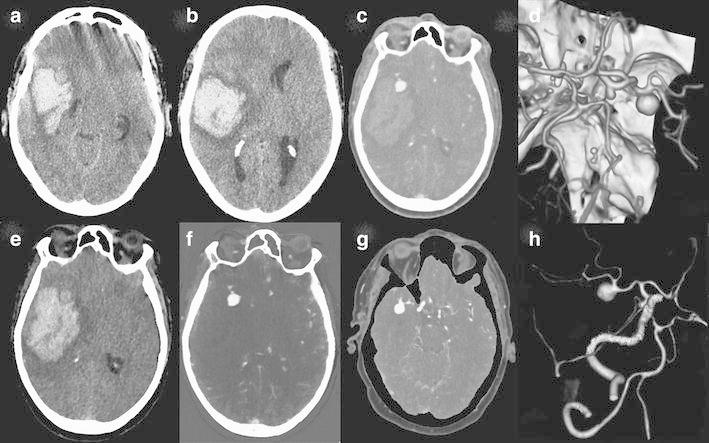
60-year-old male presented with right temporal lobe intracranial hemorrhage. Initial NECT (*a*, *b*) showed large ICH with mass effect. Mixed images (*c*) of DECTA revealed a right middle cerebral artery (MCA) aneurysm. VNC (*e*) shows the hematoma and the denser aspect of the aneurysm as also is present on the NECT. At the IOM (*f*) the aneurysm is appreciated, but not the hematoma. (*g*) shows bone-removed (BR) DECTA. VR CTA of Willis before BR (*d*) and after BR (*h*) depicts MCA aneurysm and related vessels. Occlusion of the left ICA is better visible on BR-VR, a second aneurysm is present at the posterior communicating artery. All images are deprived from one dataset. DECTA 80/Sn140 kVp, 310/155 mAs, CTDI 26.37 mGy, DLP 454 mGycm; 95 cc iodinated contrast 300 mg/ml; injection rate 5.5 cc/s; 40 cc saline flush 5.5 cc/s

**Fig. 3:**
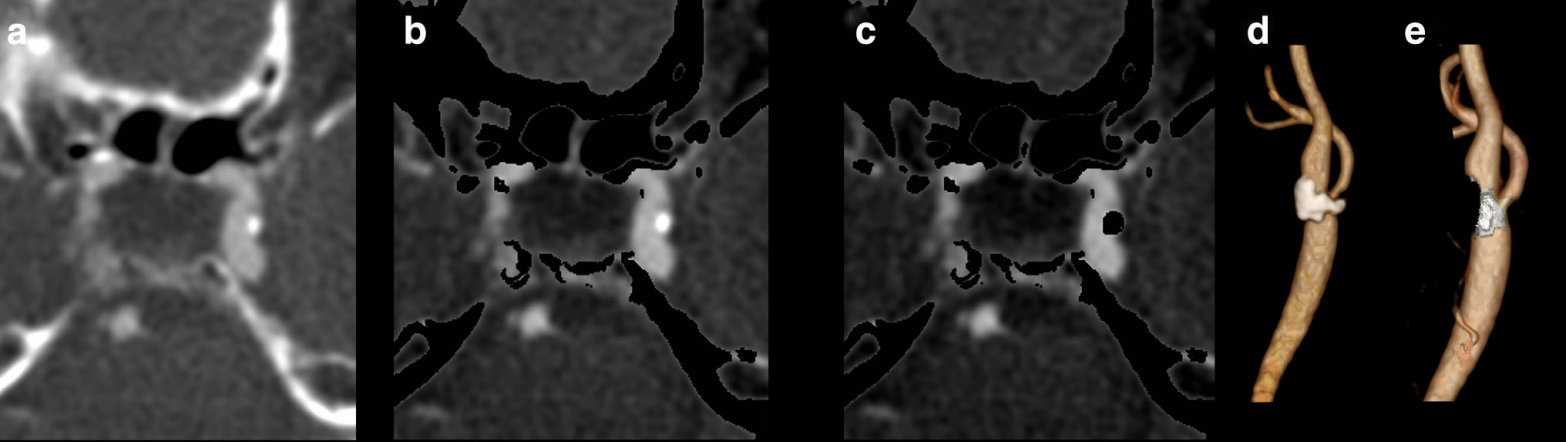
Detailed images of DECTA (*a*) at the cavernous part of the internal carotid artery (ICA), bone-removed DECTA with calcifications (*b*) and bone-removed DECTA without calcifications (*c*). Volume rendered images of carotid bifurcation with (*d*) and without (*e*) calcified plaques. DECTA 100/Sn140 kVp, 95/96 mAs, CTDI 5.9 mGy, DLP 211 mGycm; 95 cc iodinated contrast 300 mg/ml; injection rate 5.5 cc/s; 40 cc saline flush 5.5 cc/s

With the introduction of DECT, a fourth method became available, allowing for bone removal based on three-material differentiation (Fig. 2). In this way, the two scans can be replaced by one. Dependent on the DECT method used, this is achieved without increase of radiation dose [23, 24]. This method is less compromised by movement artifacts and again is non-threshold dependent. Additionally, it is possible to hide or highlight calcified plaques (plaque removal) [28, 29] (Fig. 3).

Several authors published the application of DECT-based bone removal in the intracranial vessels. Sometimes comparing DECTA with gold-standard DSA, sometimes to conventional CTA, or with bone-removed CTA [22, 24, 25, 27, 44].

Watanabe et al. compared bone-removed DECTA and CTA with the gold-standard (DSA) in a small patient group and concluded that bone removal in DECTA was good in most cases. Visualization of aneurysms next to the skull base and calcified aneurysms was superior to conventional CTA. DECTA was superior to conventional CTA for the detection and quantification of internal carotid artery (ICA) stenosis, mainly attributed to the presence of calcifications of the intracavernous and paraclinoid region of the ICA [25]. However, bone-removed DECTA lead to an overestimation of the degree of stenosis compared to DSA, due to the blooming effect of the calcifications.

Zhang et al. compared DECTA with digital subtraction CTA and 3D DSA for the detection of intracranial aneurysms in 46 and 80 patients, respectively [30, 44]. They stated that image quality of DECTA was not different from digital subtraction CTA, but at a lower radiation dose. Diagnostic accuracy of DECTA, compared to DSA, was not different for the detection of intracranial aneurysms, visualization of aneurysm morphology, and prediction of treatment (coiling vs surgery) [44]. DECTA showed a sensitivity of 97 % and a specificity of 100 % for aneurysm detection on a per-patient basis, compared to DSA.

Morhard et al. compared conventional automated bone removal with DE-based bone removal. They found that vessel delineation was better with DECT, resulting in faster reading times and a dose reduction of 29–43 % [22].

Buerke et al. compared DECTA with bone removal to time-of-flight (TOF)-MRA and standard CTA; they concluded that standard CTA and TOF-MRA were comparable, but DECTA overestimated stenosis quantification in the C2 segments, in close relationship to the bony structures. In all other segments, all three methods were similar [27].

Korn et al. investigated the grading of carotid artery stenosis in the presence of extensive calcifications by comparing DECTA and CE-MRA with the gold standard (DSA). DECTA-MPR had slightly better agreement in measuring the degree of stenosis in ACI than CE-MRA [45].

**Fig. 4:**
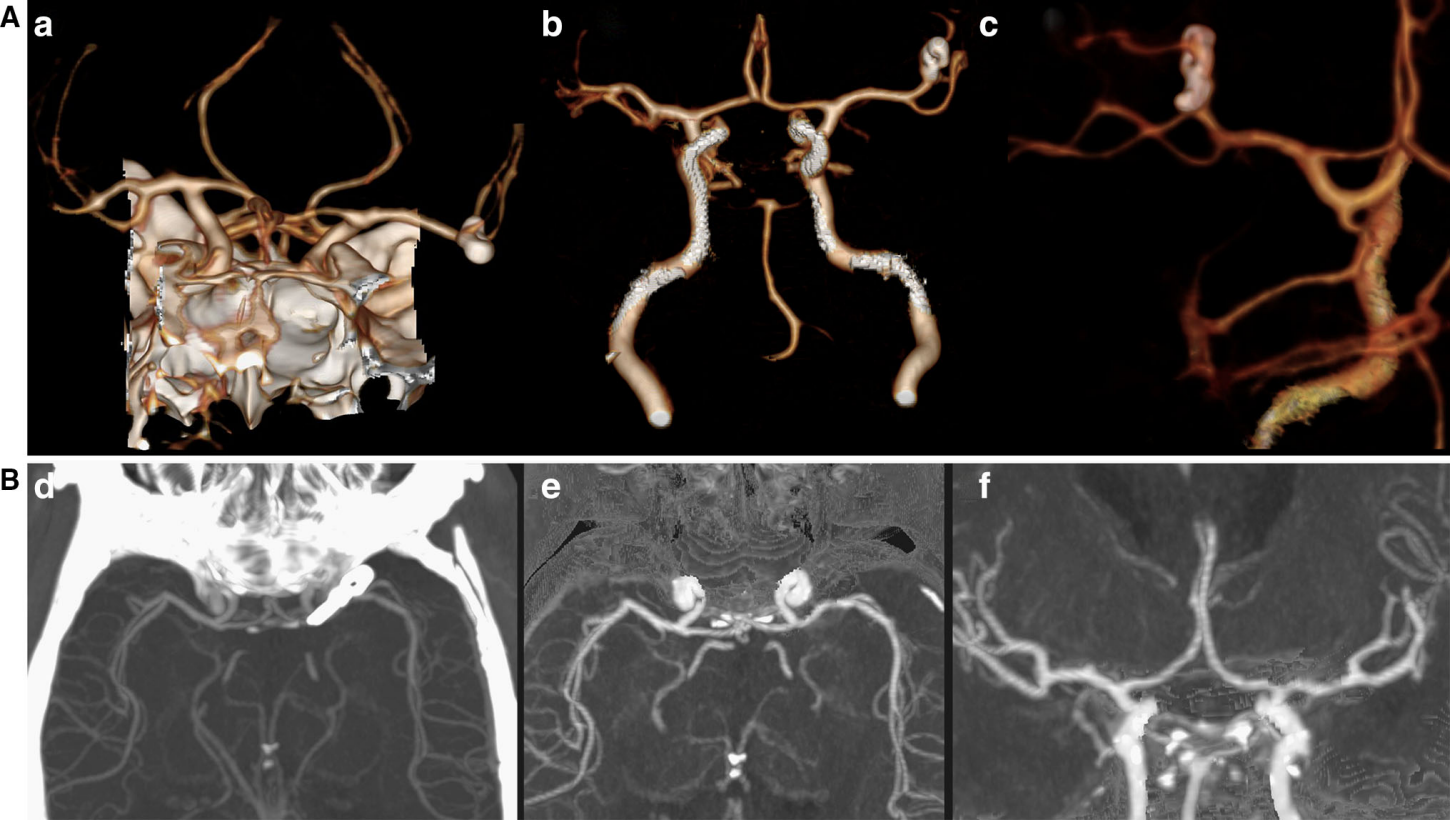
DECTA of 50-year-old male after clipping left MCA aneurysm before (*a*) and after automated bone removal (*b*, *c*—detail). The surgical clip was not removed by the automated bone removal. DECTA 80/Sn140 kVp, 310/155 mAs, CTDI 26.34 mGy, DLP 467 mGycm; 95 cc iodinated contrast 300 mg/ml; injection rate 5.5 cc/s; 40 cc saline flush 5.5 cc/s. DECTA of 51-year-old male after clipping of left carotid aneurysm before (*d*) and after automated bone removal (*e*, *f*). The surgical clip is removed by the automated bone removal. DECTA 80/Sn140 kVp, 310/155 mAs, CTDI 26,34 mGy, DLP 366 mGycm; 95 cc iodinated contrast 300 mg/ml; injection rate 5.5 cc/s; 40 cc saline flush 5.5 cc/s

Evaluation of the presence and configuration of aneurysms and surgically treated aneurysms are facilitated by DECTA [23] as the clip can be removed by DECTA post-processing, depending on the clip material used (Fig. 4).

#### Bone Removal CTA and CTV

**Fig. 5:**
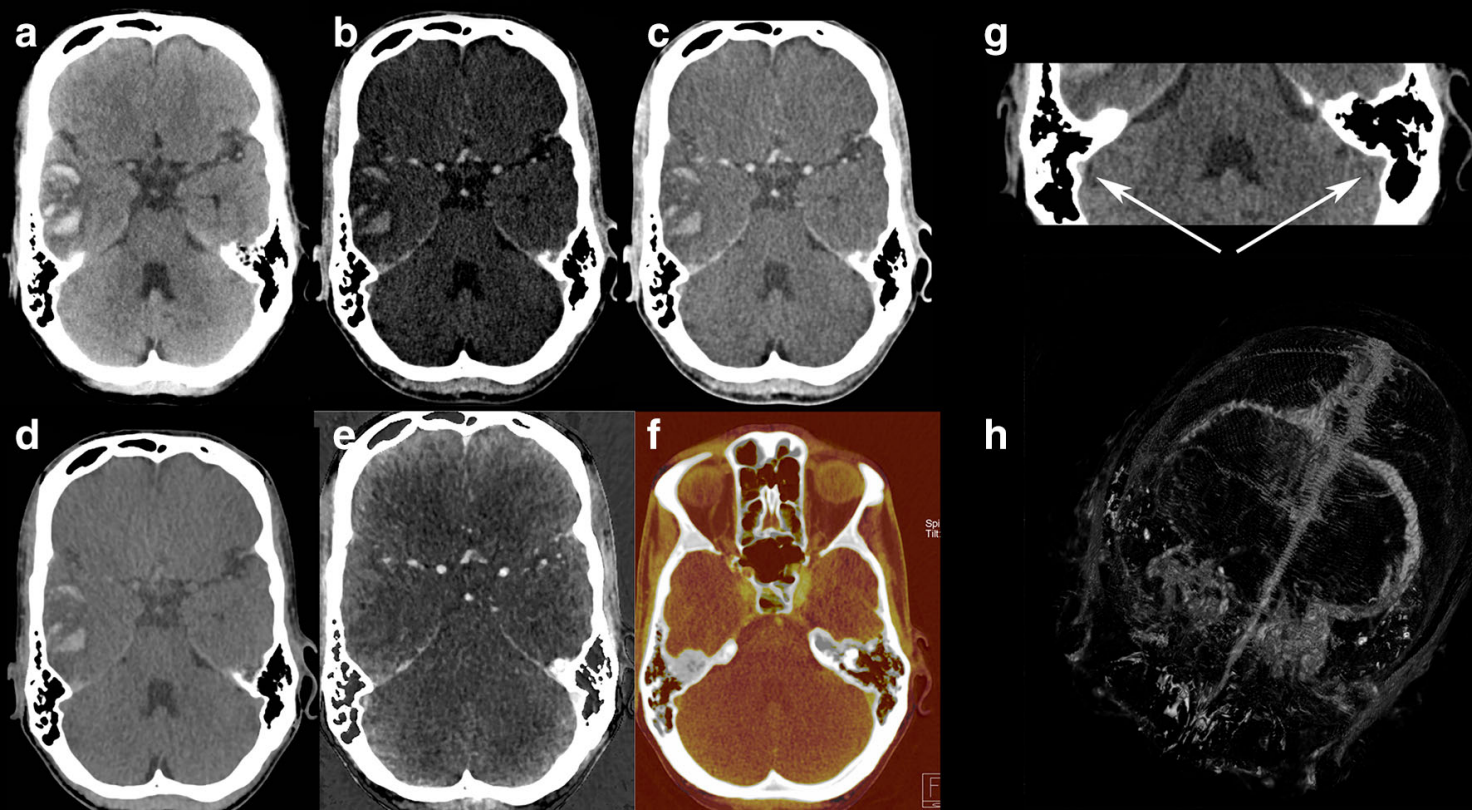
45-year-old male presented with a right temporal hemorrhage, shown at NECT (*a*). A DECTV was done. Mixed images at H31 (*b*) and H20 kernel (*c*) demonstrated bilateral density of the sigmoid sinus. VNC (*e*) and detailed VNC (*d*) demonstrates right-sided dense vessel sign, a known pitfall in CTV. IOM (*f*) and fusion (*g*) images show only left-sided enhancement of the sigmoid sinus. BR-MIP (*h*) confirms absent right sigmoid sinus, consistent with cerebral venous thrombosis. Dual energy can be helpful, especially in the case of dense thrombus (NECT 120 kVp, 285 mAs, CTDI 48.28 mGy, DLP 833 mGycm. DECTV 80/Sn140 kVp, 310/155 mAs, CTDI 26.34 mGy, DLP 451 mGycm)

In the diagnostic work-up of a suspected cerebral sinus thrombosis, CTV is one of the diagnostic options. As with CTA, MPR and thin-slab MIP are useful. After bone removal, 3D MIP and VR images can be reconstructed and easily evaluated as a 3D MIP MR venography [46]. Bone-removed DECTV is a fast and robust technique for 3D evaluation of the venous cerebral system (Fig. 5).

In conclusion, DECTA is a powerful tool for evaluation of the intracranial vessels and presence of vascular malformations like aneurysms. DECTA is equal or superior to CTA for the detection of aneurysms and is superior to CTA for the estimation of vessel integrity in the presence of calcifications.

However, DECTA tends to overestimate the degree of stenosis in comparison to DSA when calcifications, particularly close to the skull base, are present [22, 27]. Blooming artifacts caused by calcifications, especially at the skull base can induce artifacts due to excessive removal of bone at these locations or cause ill-defined borders. This underlines the necessity (as with all post-processing methods) to check the source images carefully.

#### Characterization of Iodine and Calculation of Virtual Non-enhanced Images and Iodine Maps

**Fig. 6:**
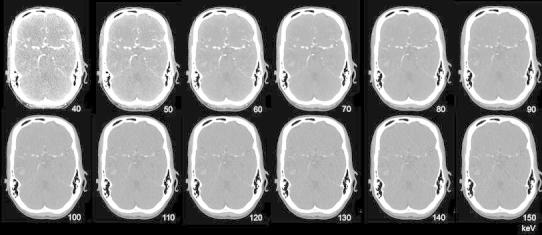
Monoenergetic images (40–150 keV) reconstructed from DECTA in a patient with right temporal hemorrhage, same as Fig. 5. An increase in iodine density at lower energies is shown. At higher energies, the hemorrhage is better appreciated, whereas the iodine attenuation decreases. CNR is higher at lower energies, while SNR increases with higher energies

Standard CT at 120 kVp exhibits similar Hounsfield densities for iodine and hemorrhage. But at lower kV, as iodine is closer to the K-edge, attenuation is increased compared to hemorrhage and iodine shows higher densities (Fig. 6). DECT is able to characterize iodine and to calculate VNC and IOM. These are useful in various neuroradiological indications.

#### After Intravenous or Intra-arterial Contrast

**Fig. 7:**
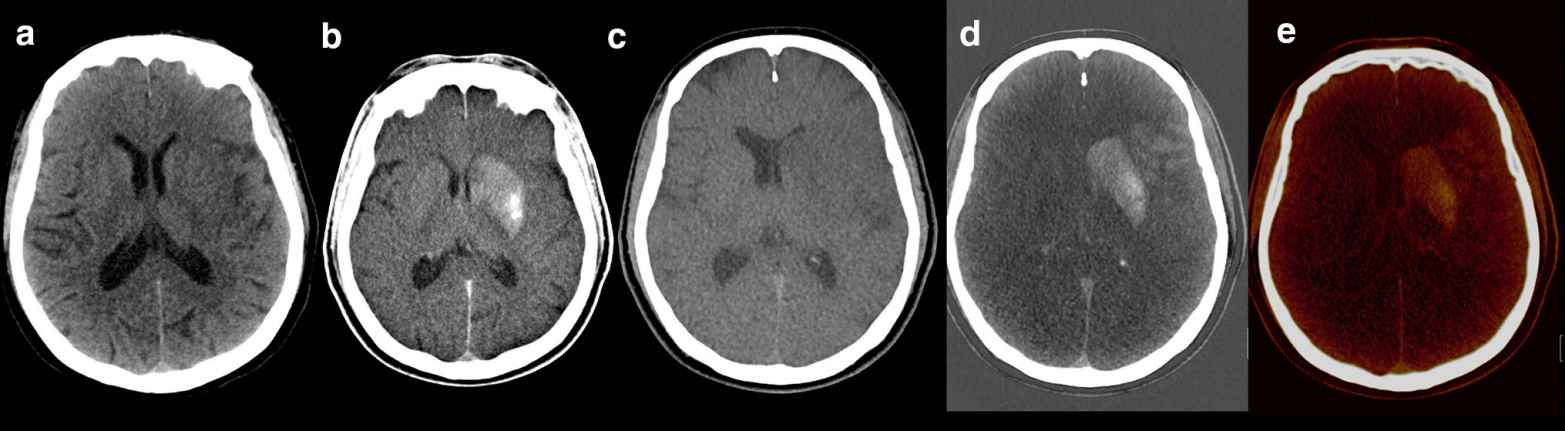
71-year-old male presented with right-sided hemiparesis due to left MCA occlusion. NECT showed faint loss of left lentiform nucleus and a left insular ribbon sign. The thrombus of the left MCA was removed by mechanical thrombectomy after stenting the left ICA. Mixed images (*b*) of DECT immediately after IAR demonstrated density in the left basal ganglia, most pronounced at the putamen. VNC (*c*) showed faint hypodensity in the left MCA territory, but no signs of hemorrhage. IOM (*d*) and fusion images (*e*) demonstrated hyperdensity in the left basal ganglia and cortex of MCA territory, consistent with blood brain barrier breakdown without hemorrhage. Anticoagulant therapy, because of stent placement, was safely initiated. Follow-up imaging confirmed infarction in part of the left MCA territory, but no signs of hemorrhage (NECT 120 kVp, 310 mAs, CTDI 47.25 mGy, DLP 874 mGycm. DECT 80/Sn140 kVp, 392/196 mAs, CTDI 36.43 mGy, DLP 666 mGycm)

In stroke patients, IAR—either by thrombolysis or by thrombectomy—are therapeutic options. A feared complication in this patient group is hemorrhage, in up to 15 % of patients [47–50]. CT immediately after IAR can be difficult to interpret, due to the afore-mentioned overlap in densities [51–53]. It has been shown that DECT has added value in this patient group (Fig. 7). Tijssen et al. demonstrated an increased accuracy of hemorrhage detection by DECT compared to mixed imaging alone in a group of 30 patients after IAR [15•]. Hyperdensities were encountered in 19 patients. When reporting mixed imaging only, comparable with standard CT, the positive predictive value (PPV) of hemorrhage detection was 25 %, with a negative predictive value (NPV) of 91 % and an overall accuracy of 63 %. The PPV for the detection of hemorrhage with DECT using IOM and VNC images was 100 %, with a NPV of 89 %, accuracy improved to 89 %. The authors concluded that the diagnostic accuracy and confidence increased in early differentiation between hemorrhage and contrast for DECT, even at a lower dose than conventional CT.

Morhard et al. found that hyperdensities at CT immediately after IAR was a frequent finding in 48 of their 60 patients [13•]. In 10.4 % (5/48), this was present on VNC and was rated as hemorrhage; in 89.6 % (43/48), this was only present on iodine images and was related to blood brain barrier breakdown (BBBB). They encountered no false positive or negative ICH compared to follow-up CT and/or MRI. They stated that DECT allows for a reliable differentiation between BBBB and ICH.

Gupta et al. studied 15 patients with high density lesions at CT after administration of intravenous or intra-arterial iodinated CM, 12 of these were scanned after IAR. They found a sensitivity of 100 % with a specificity of 91 % for the identification of hemorrhage, with an accuracy of 93 % [19].

Phan et al. used DECT in a group of 40 patients after contrast administration. Indications were evaluation of carotid stenosis, trauma, tumors, and after IAR. Of 148 hyperdensities on CT, DECT correctly classified 142 lesions as hemorrhage or iodine. Sensitivity was 100 %, specificity 84–100 %, and accuracy 87–100 %, depending on the location of the density (parenchymal, subarachnoid, or intraventricular). Radiation dose in their study was comparable with a conventional CT (CTDI 66 mGy). The iodine map and VNC derived from DECT were noisier than mixed images but were rated sufficient for diagnosis. In their study, calcifications (as in the pineal gland) were a confounder and could not be discriminated from iodine. The latter is confirmed by Dinkel et al. who concluded that the presence of a fourth material cannot be depicted by the three-material decomposition algorithm and will be misclassified [54•].

Brockman et al. used DECT after peri-interventional subarachnoid hemorrhage SAH. Allowing for discrimination and subtraction of blood and iodine mixed within the subarachnoid space in patients with peri-interventional re-SAH. They stated that overestimation of SAH is reduced after peri-interventional rebleeding and is therefore valuable [26].

#### Intracranial Hemorrhage: Identification Vascular Malformation, Tumor, and Spot Sign

**Fig. 8:**
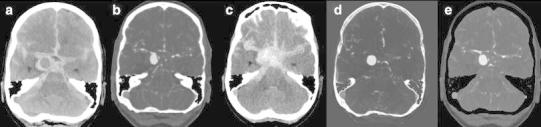
61-year-old female presented with a SAH due to ruptured carotid aneurysm. NECT (*a*) demonstrated the profound SAH. Mixed images at H20 kernel (*b*) revealed the carotid aneurysm. SAH is easily depicted at the VNC (*c*), at lower SNR, but higher CNR, while the aneurysm is depicted at the IOM (*d*). Same image as (*b*) after BR (*e*) (NECT 120 kVp, 285 mAs, CTDI 48.28 mGy, DLP 666 mGycm. DECT 80/Sn140 kVp, 310/155 mAs, CTDI 26.34 mGy, DLP 457 mGycm; 95 cc iodinated contrast 300 mg/ml; injection rate 5.5 cc/s; 40 cc saline flush 5.5 cc/s)

**Fig. 9:**
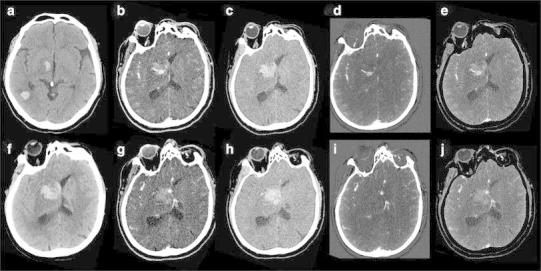
65-year-old male presented with left-sided hemiparesis due to ICH. The patient deteriorated within 1 h and a DECTA/DECTV was performed. The patient was non-cooperative at DECTA as demonstrated by the rotated head. NECT (*a*—at presentation, *f*—1 h later), mixed images (H31 kernel) (*b*, *g*), VNC (*c*, *h*), IOM (*d*, *i*), and bone removal (*e*, *j*) from DECTV are shown. The hematoma is expanded in between the two conventional CT scans. Two extravascular enhancing lesions are present at the mixed, IOM, and bone removal images, consistent with spot signs. The VNC clearly demonstrates the hematoma at lower SNR but still rated as diagnostic. All reconstructions, even bone removal, from DECTA can be made, despite the relative non-cooperation of the patient (conventional CT (*a*) 120 kVp, 310 mAs, CTDI 69.44 mGy, DLP 1062 mGycm. Conventional CT (*f*) 120 kVp, 285 mAs, CTDI 48.28 mGy, DLP 833 mGycm. DECTV 80/Sn140 kVp, 310/155 mAs, CTDI 28.86 mGy, DLP 520 mGycm; 95 cc iodinated contrast 300 mg/ml; injection rate 5.5 cc/s; 40 cc saline flush 5.5 cc/s, delay 45 s)

In patients presenting with ICH, CTA is performed for detection of aneurysms in case of SAH. In theory NECT and CTA could be replaced by DECTA with VNC (Figs. 8, 9). Ferda assessed 25 patients presenting with intracranial bleeding on NECT and performed DECTA. All patients had SAH, where in 10/25 patients, this was combined with intraventricular, intracerebral, and/or subdural bleeding [31]. They assessed the diagnostic value of VNC imaging obtained from DECTA, as well as image quality and CNR between bleeding and hemorrhage. They concluded that VNC images would be sufficient for the detection of hemorrhage in DECTA with a per-patient accuracy of 100 % and a lesion detection of 96 %. The CNR was lower in DECTA compared to NECT, but still rated as sufficient by the readers. The authors conclude that by omitting the unenhanced CT in this patient group in the future, radiation can be reduced. In clinical practice, this only applies to a small patient group.

**Fig. 10:**
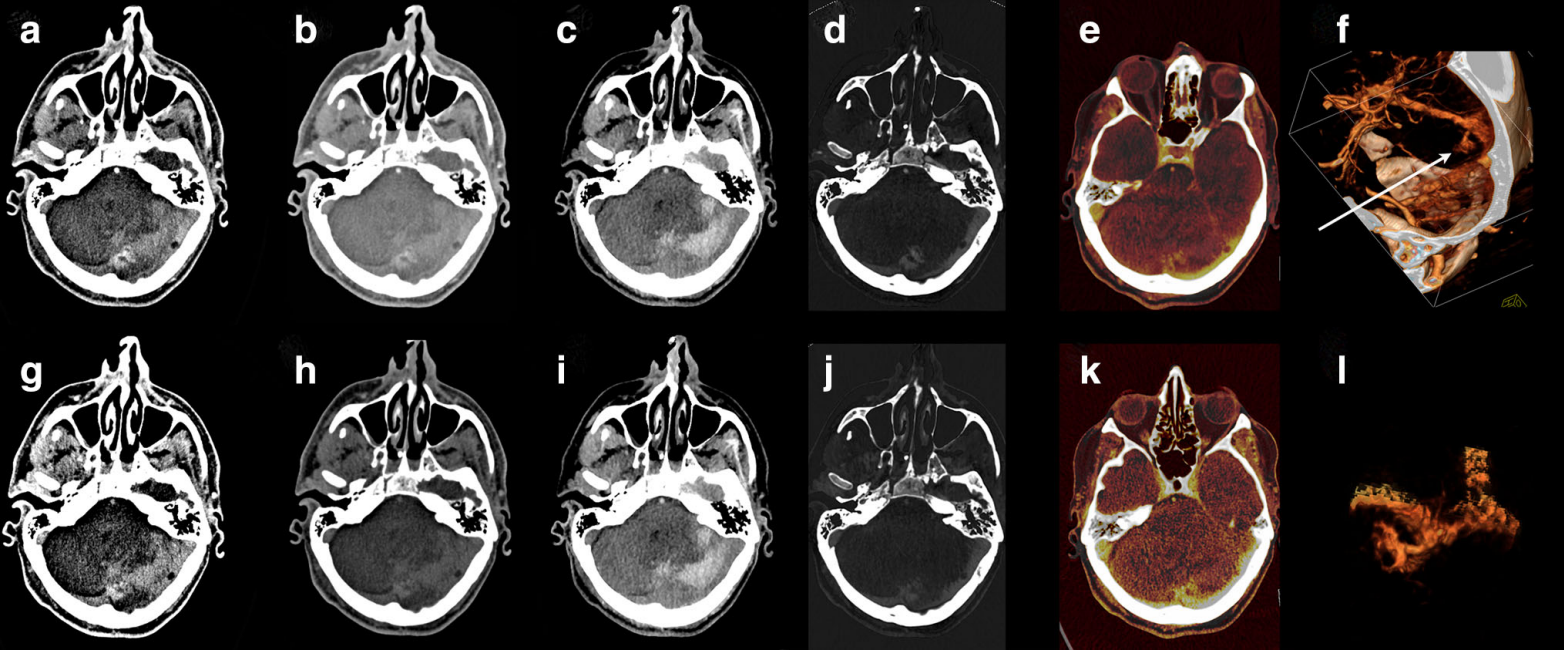
55-year-old male presented with a posterior fossa hemorrhage for which decompression was performed. After surgery DECTA and DECTV were made in search of underlying pathology. Shown are CTA: mixed images with H31 kernel (*a*), mixed images with H20 kernel (*b*), VNC (*c*), IOM (*d*), fusion images (*e*) and manually segmented VR from IOM (*f*, *l*). The lower series shows DECTV: mixed images H31 kernel (*g*), mixed images H20 kernel (*h*), VNC (*i*), IOM (*j*), fusion images (*k*). A residual hematoma is found in the posterior fossa. A developmental venous anomaly (DVA) is shown, best depicted on IOM and fusion images in the arterial phase. VR images from iodine images clearly demonstrate the DVA (*arrow*). This was not visible when VR was done from mixed source images of DECTA/DECTV (not shown) (DECTA 80/Sn140 kVp, 310/155 mAs, CTDI 26.34 mGy, DLP 575 mGycm DECTV 80/Sn140 kVp, 310/155 mAs, CTDI 26.34 mGy, DLP 576 mGycm; 95 cc iodinated contrast 300 mg/ml; injection rate 5 cc/s; 40 cc saline flush 5 cc/s)

**Fig. 11:**
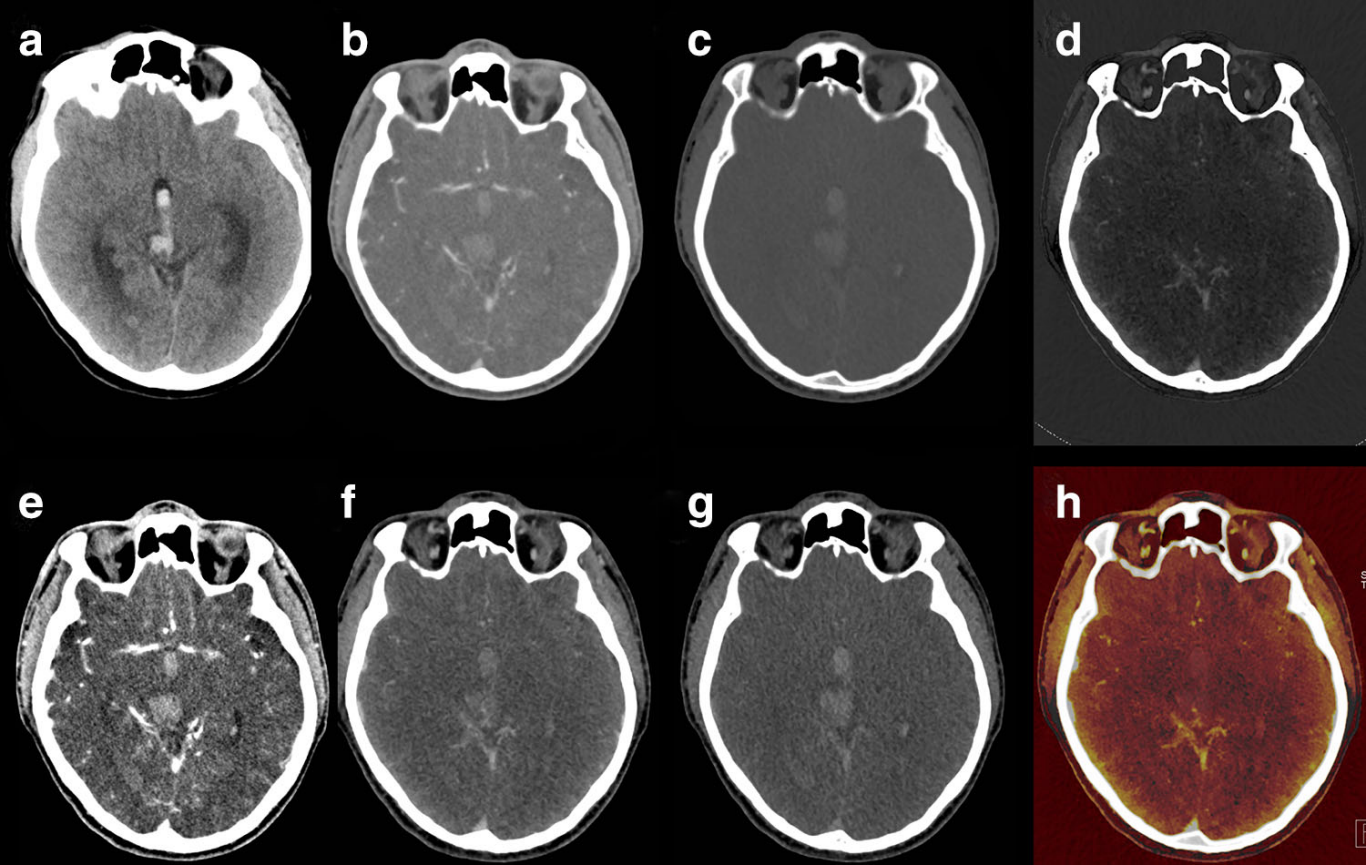
55-year-old male presented with midbrain hemorrhage. DECTA was subsequently performed. Shown are NECT (*a*), mixed images DECTA H20 kernel (*b*), VNC (*c*), IOM (*d*), mixed images DECTA H31 kernel (*e*), 80 kVp images (*f*), 140 kVp images (*g*) and fusion images (*h*). NECT at presentation showed a small mesencephalic hemorrhage. DECTA mixed images at H20 (*b*) and H31 (*e*) kernels did not show any underlying abnormalities. However, a small vascular malformation was appreciated at the IOM (*d*), 80 kVp (*f*) and fusion images (*h*), clearly demonstrating the additive value of DECT reconstructions (conventional CT 120 kVp, 285 mAs, CTDI 48.28 mGy, DLP 833 mGycm. DECTA 80/Sn140 kVp, 310/155 mAs, CTDI 26.34 mGy, DLP 475 mGycm; 95 cc iodinated contrast 300 mg/ml; injection rate 5.5 cc/s; 40 cc saline flush 5.5 cc/s)

**Fig. 12:**
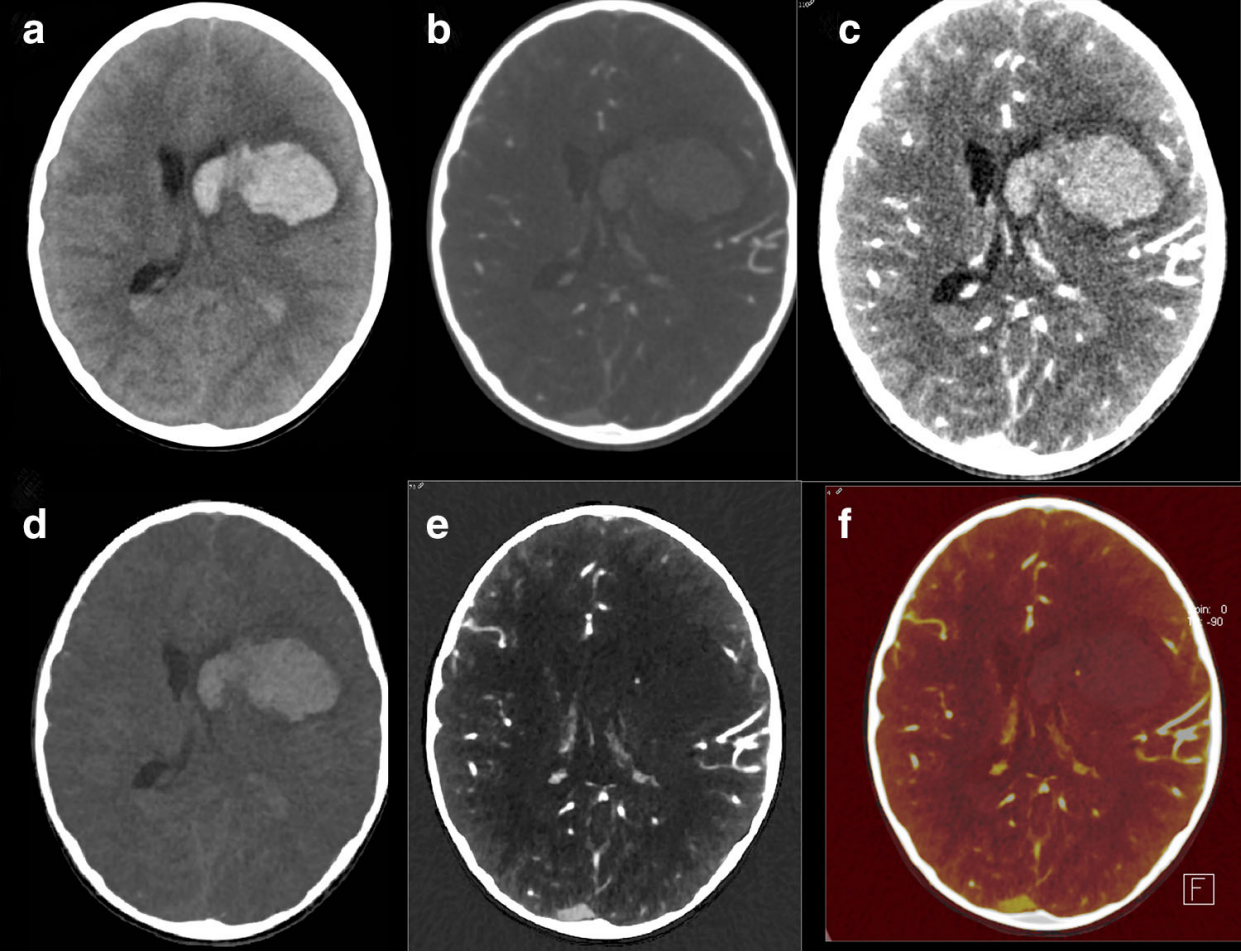
10-year-old female presented with right-sided hemiparesis based on ICH within the left hemisphere with intraventricular extension shown at NECT (*a*). A DECTA was performed. Shown are mixed images at H20 (*b*) and H31 kernel (*c*), without underlying pathology and initially read as without spot sign. VNC (*d*) visualized the hematoma. IOM (*e*) and fusion images (*f*) clearly demonstrated a spotsign centrally in the hematoma (conventional CT 120 kVp, 300 mAs, CTDI 44.47 mGy, DLP 747 mGycm. DECTA 80/Sn140 kVp, 310/155 mAs, CTDI 26.34 mGy, DLP 433 mGycm). 50 cc iodinated contrast 300 mg/ml; injection rate 2.8 cc/s; 20 cc saline flush 2.8 cc/s)

CTA, CT perfusion, and CTV are increasingly used for identification of spot signs and identification of underlying tumor [39, 55]. Up to 30 % of ICH is secondary to vascular malformation or tumor (Figs. 10, 11) [56]. The presence of active contrast extravasation in an intracerebral hemorrhages is associated with an increased risk of hematoma expansion [55, 57, 58]. Detection of spot sign can be very difficult, especially in high density intracerebral hemorrhage. The detection of the spot sign can be improved by increasing the arterial scan delay with 10 s [59], delayed imaging [60] or dynamic imaging [61, 62], and with DECT (Fig. 12). Won et al. stated that DECT in an animal model allowed for quantifying iodine as marker for ongoing bleeding in anti-coagulation associated ICH [63].

Watanabe et al. performed a retrospective study in 36 patients presenting with acute intracranial hematomas [16••]. They performed a NECT and a DECTA and compared the latter with standard CT images with delayed enhancement. The patient population consisted of 16 idiopathic ICH, 16 traumatic ICH, 2 patients with SAH combined with ICH, and two patients with intra-tumor hemorrhage. They evaluated the combined images of DECTA, simulating 120 kVp and iodine images with late enhancement standard CT images. In 57, delayed enhancement in the hemorrhage was seen. Using the iodine images compared to combined imaging, sensitivity, negative, and PPVs of contrast leakage or enhancement increased with constant specificity (95.7/82.6 %; 95.7/95 %; 94.1/80 %; and 94.1/94.1 %, respectively). The authors found three lesions in the iodine images that could not be detected on combined imaging alone, and in four patients, enhancement of lesions was better depicted on the iodine images, compared to combined imaging. They concluded that DECT emphasized iodine enhancement and facilitated detection of contrast enhancement or leakage.

Kim et al. evaluated 56 patients, presenting with intracerebral hematoma with DECT to differentiate primary ICH from tumor bleeding [39]. They evaluated the accuracy of mixed images, mixed images combined with true non-enhanced (TNC) images and fusion images in detection of underlying tumors. Of 18 tumorous lesions, they identified 17 with fusion imaging, 12 on mixed images combined with TNC, and 11 on mixed images alone. They encountered 1 false positive with fusion images, which turned out to be a cavernoma. They concluded that the diagnostic performance of fusion imaging was higher than that of mixed imaging, whether or not combined with a TNC. Sensitivity and specificity of fusion images were 94.4 and 97.4 %, respectively. There was a significant difference with mixed imaging (sens. 61.1 % and spec. 92.3 %; *p* = 0.006) and with mixed imaging/TNC (sens. 66.7 %; spec. 89.7 %; *p* = 0.011). The image quality of TNC images was superior to VNC images, but the latter had lower noise in the measurements. CNR was higher in VNC images compared to TNC. Although MRI is known to be superior in detection of underlying tumor in ICH, this is known to be difficult in the acute stage. DECT can play an important role in the acute and subacute stage, thereby enabling early diagnosis [39].

## Virtual Monoenergetic Imaging/Reconstructions

When scanned in DE mode, monoenergetic or monochromatic images can be reconstructed. As in polychromatic CT with increasing (tube) voltage, the SNR increases while tissue contrast decreases. Lowering the tube voltage will increase the CNR while the SNR decreases. DECT takes advantage of both [63–65].

### Increased Attenuation of Iodine

In cerebral CTA, virtual monoenergetic imaging can increase vascular enhancement by increasing CNR at lower virtual monoenergetic images [66, 67] (Fig. 6). The increase of vascular enhancement provides opportunities to lower the amount of iodine [32, 68]. Schneider et al. determined the optimal settings in cerebral and cervical DECTA with monoenergetic imaging [14]. They found an optimum vessel attenuation and CNR for cerebral CTA at 60 keV when compared to 120 kVp polychromatic imaging.

### Optimized Brain Imaging

**Fig. 13:**
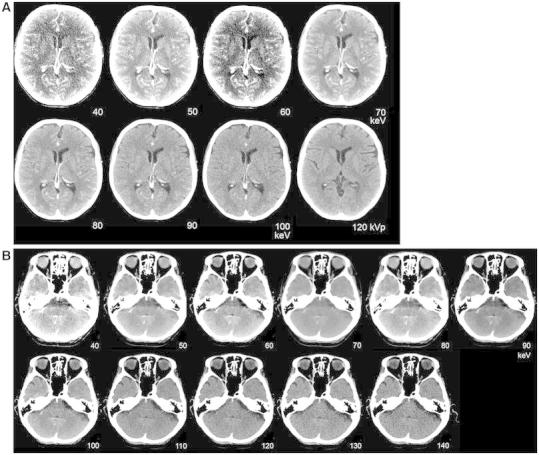
Monoenergetic reconstructions derived from a DECTV at the level of the supratentorial white matter/basal ganglia and the posterior fossa. SECT at 120 kVp is added for comparison. At lower monoenergies the SNR is lower, with higher CNR. Optimum level in the supratentorial brain is between 50 and 70 keV. At lower energies the beam-hardening artifacts in the posterior fossa are more pronounced, whereas they decrease at higher energies. Optimal imaging requires higher energies for posterior fossa compared to supratentorial brain (conventional CT 120 kVp, 285 mAs, CTDI 48.28 mGy, DLP 666 mGycm. DECTA 80/Sn140 kVp, 310/155 mAs, CTDI 28.86 mGy, DLP 458 mGcm. 90 cc iodinated contrast 300 mg/ml; injectionrate 3 cc/s; 40 cc saline flush 3 cc/s, delay 45 s)

Monoenergetic images can be used to optimize brain visualization in non-enhanced scans (Fig. 13). Pomerantz et al. [69••] determined the optimal virtual monochromatic imaging levels that maximized brain parenchymal image quality in non-enhanced DECT of the brain in 75 patients, excluding patients with intracranial hardware. They concluded that the maximal SNR and CNR for supratentorial gray and white matter was at 65 keV monoenergetic energy level. The optimum energy level regarding artifacts in the posterior fossa was at 75 keV. At this, elevated keV beam-hardening artifacts in the posterior fossa were reduced. The use of these variable keV images showed improved image quality compared to 120 kVp polychromatic CT.

Shinohara et al. used monoenergetic imaging in CTA to reduce artifacts surrounding platinum coils. They used high keV and additional metal artifacts reduction software. At increasing keV, the SNR increased and metallic artifacts were reduced by reducing beam-hardening artifacts [34].

### Spine Imaging

Next to brain imaging, DECT can be useful in spinal imaging, especially when metallic implants are present. In patients with metallic implants, like in spinal fusion implants, imaging of the spine and spinal canal can be challenging with CT as well as MRI [70]. It is applicable to identify complications of the hardware, like malpositioning, loosening, and disruption. In patients with contra-indications for MRI or inability to perform MRI due to susceptibility artifacts, CT myelography is an alternative for spine imaging. The presence of metal leads to beam-hardening and streak artifacts due to photon starvation. Possible solutions to improve image quality are using high kVp, reduced voxel size, oversampling, and high definition scanning. Increasing the kVp reduces beam-hardening artifacts, even though at the price of increased radiation dose. Iterative reconstruction techniques improve image quality by reducing artifacts, as well as dose reduction [70].

**Fig. 14:**
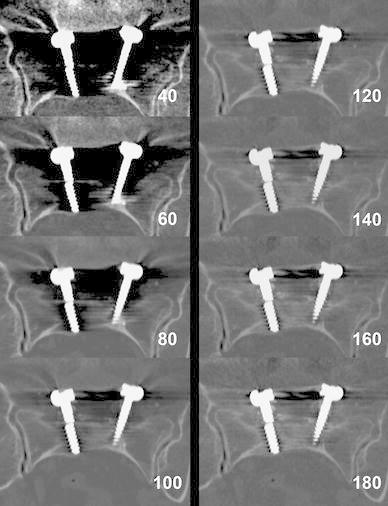
Monoenergetic reconstructions from DECT dataset of a 67-year-old female with lumbar spondylodesis. Shown are reconstructions from 40 to 180 keV showing a right-sided broken screw. Increasing keV allows better visualization of the screws with improved hardware-bone interface and decreased beam-hardening artifacts (DECT 100/Sn140 kVp, 250/483 mAs, CTDI 31.03 mGy, DLP 682 mGycm.)

Application of DECT, especially monoenergetic reconstruction, in spinal imaging is published by several authors. Monoenergetic imaging with DECT reduces the amount of metallic artifacts by decreasing beam-hardening artifacts and increases image quality at higher monoenergetic keV levels, improving the assessment of screws and hardware-bone interface (Fig. 14).

Srinivasan described the use of DECT virtual monochromatic series for assessment of spinal transpedicular hardware-bone interface in 92 patients [35]. They concluded that monochromatic images at 110 keV or higher were beneficial in the reduction of metallic streak artifacts and enabled better visualization of the hardware-bone interface than 70 keV (comparable with 120 kVp standard CT) series in patients with spinal transpedicular screw fixation. Guggenberger et al. evaluated the optimal monoenergetic DECT settings for artifact reduction in posterior spine implants from different vendors in a phantom [33]. They demonstrated that monoenergetic DECT images provided better image quality and less metallic artifacts than standard CT. They showed that image quality was superior at higher monoenergies of DECT with artifact reduction at higher energies, with an optimum at 120–140 keV, dependent on vendor and spine level. They concluded that keV values in monoenergetic reconstructions should be individually tailored dependent on brand of implants and spine-level characteristics [33].

Wang et al. evaluated monochromatic images in pedicle screws in patients with scoliosis and concluded that at higher monochromatic photon energy image quality was increased compared to polychromatic imaging, and more accurate measurements of the width of the screws were obtained. They found an optimum range for reducing metal artifacts at 110–140 keV [71].

DECT myelography can be a solution in patients with spinal instrumentation [65]. Grams et al. demonstrated that DECT myelography after lumbar osteosynthesis with DECT provided minimal artifact disturbance by reducing beam-hardening artifacts using the tube voltage of 140 kVp, compared to 80 and 120 kVp blended images. Virtual myelography was superior to conventional myelography in their group of 30 patients.

DECT myelography can be an opportunity to reduce dose compared to standard CT. The use of monoenergetic reconstructions can minimize beam-hardening artifacts, and application of material decomposition offers the possibility of virtual myelography [65].

### Future Developments and Challenges

DECT in neuroradiology is catching up with other radiological subspecialties. The number of neuroradiological publications is steadily increasing and its strength is recognized. This encourages further refinement of DECT algorithms and applications in brain imaging and asks for solution of the limitations in DECT, as the confounding fourth element in three-material decomposition.

Recently, advanced monoenergetic reconstructions were introduced, combining the advantages of low and high keV reconstructions using only a part of the frequency spectrum of both. This has the potential to put monoenergetic reconstructions to a higher quality level.

The use of iterative reconstructions in DECT can improve SNR, especially in non-full dose VNC and IOM.

## Conclusion

DE scanning in neuroradiology does clearly have an added value. Bone removal, material characterization, and monoenergetic reconstructions allow for improved detection of aneurysms, vascular malformations, and iodine enhancement all within one scan. Monoenergetic reconstructions help improving image quality of the brain and spine and/or improved iodine attenuation.

When material characterization is desired, DECT offers more information at the same image quality while maintaining dose neutrality.

## Abbreviations

BBBB: Blood brain barrier breakdown
BR: Bone removed
CM: Contrast media
CT: Computed tomography
CTA: CT angiography
CTV: CT venography
CNR: Contrast-to-noise ratio
DE: Dual energy
DECT: Dual-energy CT
DECTA: Dual-energy CTA
DECTV: Dual-energy CT venography
DSA: Digital subtraction angiography
IAR: Intra-arterial recanalization/revascularization
ICA: Internal carotid artery
ICH: Intracranial hemorrhage
IOM: Iodine map
keV: Kilo electron voltage
kVp: Peak kilo voltage
MDCT: Multidetector-row CT
MCA: Middle cerebral artery
MRI: Magnetic resonance imaging
MRV: MR venography
MIP: Maximum intensity projection
MPR: Multiplanar reformation
SNR: Signal-to-noise ratio
TNC: True non-contrast
TOF: Time-of-flight
VNC: Virtual non-contrast
VR: Volume rendering

## References

[1] Hoeffner EG, Mukherji SK, Srinivasan A, Quint DJ (2012). Neuroradiology back to the future: spine imaging. AJNR Am J Neuroradiol.

[2] Hoeffner EG, Mukherji SK, Srinivasan A, Quint DJ (2012). Neuroradiology back to the future: brain imaging. AJNR Am J Neuroradiol.

[3] Brooks RA (1977). A quantitative theory of the Hounsfield unit and its application to dual energy scanning. J Comput Assist Tomogr.

[4] Flohr TG, McCollough CH, Bruder H (2006). First performance evaluation of a dual-source CT (DSCT) system. Eur Radiol.

[5] Bautz W, Kalender WA (1987). Material selective imaging and determination of density using the dual energy method. II. Clinical use of dual energy radiography. Digitale Bilddiagn.

[6] Kalender W, Bautz W, Felsenberg D, Suss C, Klotz E (1987). Material-selective imaging and density measurement using the dual-energy method. I. Principles and methodology. Digitale Bilddiagn.

[7] Tawfik AM, Kerl JM, Bauer RW (2011). Dual-energy CT of head and neck cancer: average weighting of low- and high-voltage acquisitions to improve lesion delineation and image quality-initial clinical experience. Invest Radiol.

[8] Tawfik AM, Kerl JM, Razek AA (2011). Image quality and radiation dose of dual-energy CT of the head and neck compared with a standard 120-kVp acquisition. AJNR Am J Neuroradiol.

[9] Tawfik AM, Razek AA, Kerl JM, Nour-Eldin NE, Bauer R, Vogl TJ (2014). Comparison of dual-energy CT-derived iodine content and iodine overlay of normal, inflammatory and metastatic squamous cell carcinoma cervical lymph nodes. Eur Radiol.

[10] Srinivasan A, Parker RA, Manjunathan A, Ibrahim M, Shah GV, Mukherji SK (2013). Differentiation of benign and malignant neck pathologies: preliminary experience using spectral computed tomography. J Comput Assist Tomogr.

[11] Toepker M, Czerny C, Ringl H (2014). Can dual-energy CT improve the assessment of tumor margins in oral cancer?. Oral Oncol.

[12] Grams AE, Sender J, Moritz R (2014). Dual energy CT myelography after lumbar osteosynthesis. Fortschr Rontgenstr.

[13] • Morhard D, Ertl L, Gerdsmeier-Petz W, Ertl-Wagner B, Schulte-Altedorneburg G. Dual-energy CT immediately after endovascular stroke intervention: prognostic implications. Cardiovasc Intervent Radiol. 2014;37:1171–178. *Evaluated the incidence of ICH and BBBB immediately after endovascular recanalization therapy. They found no correlation between BBBB and infarct size. No false positive and false negative ICH were found when DECT was used for differentiation of ICH and BBBB*. 10.1007/s00270-013-0804-y 24310826

[14] Schneider D, Apfaltrer P, Sudarski S (2014). Optimization of kiloelectron volt settings in cerebral and cervical dual-energy CT angiography determined with virtual monoenergetic imaging. Acad Radiol.

[15] • Tijssen MP, Hofman PA, Stadler AA et al. The role of dual energy CT in differentiating between brain hemorrhage and contrast medium after mechanical revascularisation in acute ischaemic stroke. 2014;Eur Radiol. 24:834–40. *Evaluated DECT in patients after IAR. They concluded that diagnostic accuracy and confidence increased in early differentiation between hemorrhage and contrast for DECT, even at lower dose than conventional CT.* 10.1007/s00330-013-3073-x 24258277

[16] •• Watanabe Y, Tsukabe A, Kunitomi Y et al. Dual-energy CT for detection of contrast enhancement or leakage within high-density haematomas in patients with intracranial hemorrhage. Neuroradiology. 2014;56:291–95. *Compared DECT with delayed enhanced conventional CT for the presence of contrast enhancement in intracranial hematomas. They found that 57.5* *% of hematomas demonstrated contrast enhancement or extravasation. They found a sensitivity, specificity, PPV and NPV of 82.6, 94.1, 95.80* *% for the combination of non-contrast CT and combined CT. This increased to 95.7, 94.1, 95.7 and 94.1* *% when non-contrast CT and combined CT were combined with iodine images*. 10.1007/s00234-014-1333-3 24510167

[17] Postma AA, Hofman PA, Stadler AA, van Oostenbrugge RJ, Tijssen MP, Wildberger JE (2012). Dual-energy CT of the brain and intracranial vessels. AJR Am J Roentgenol.

[18] Vogl TJ, Schulz B, Bauer RW, Stover T, Sader R, Tawfik AM (2012). Dual-energy CT applications in head and neck imaging. AJR Am J Roentgenol.

[19] Gupta R, Phan CM, Leidecker C (2010). Evaluation of dual-energy CT for differentiating intracerebral hemorrhage from iodinated contrast material staining. Radiology.

[20] Phan CM, Yoo AJ, Hirsch JA, Nogueira RG, Gupta R (2012). Differentiation of hemorrhage from iodinated contrast in different intracranial compartments using dual-energy head CT. AJNR Am J Neuroradiol.

[21] Johnson TR, Krauss B, Sedlmair M (2007). Material differentiation by dual energy CT: initial experience. Eur Radiol.

[22] Morhard D, Fink C, Becker C, Reiser MF, Nikolaou K (2008). Value of automatic bone subtraction in cranial CT angiography: comparison of bone-subtracted vs. standard CT angiography in 100 patients. Eur Radiol.

[23] Hegde A, Chan LL, Tan L, Illyyas M, Lim WE (2009). Dual energy CT and its use in neuroangiography. Ann Acad Med Singapore.

[24] Morhard D, Fink C, Graser A, Reiser MF, Becker C, Johnson TR (2009). Cervical and cranial computed tomographic angiography with automated bone removal: dual energy computed tomography versus standard computed tomography. Invest Radiol.

[25] Watanabe Y, Uotani K, Nakazawa T (2009). Dual-energy direct bone removal CT angiography for evaluation of intracranial aneurysm or stenosis: comparison with conventional digital subtraction angiography. Eur Radiol.

[26] Brockmann C, Scharf J, Nolte IS, Seiz M, Groden C, Brockmann MA (2010). Dual-energy CT after peri-interventional subarachnoid hemorrhage: a feasibility study. Clin Neuroradiol.

[27] Buerke B, Puesken M, Wittkamp G (2010). Bone subtraction CTA for transcranial arteries: intra-individual comparison with standard CTA without bone subtraction and TOF-MRA. Clin Radiol.

[28] Ma R, Liu C, Deng K, Song SJ, Wang DP, Huang L (2010). Cerebral artery evaluation of dual energy CT angiography with dual source CT. Chin Med J.

[29] Muhlenbruch G, Das M, Mommertz G (2010). Comparison of dual-source CT angiography and MR angiography in preoperative evaluation of intra- and extracranial vessels: a pilot study. Eur Radiol.

[30] Zhang LJ, Wu SY, Niu JB (2010). Dual-energy CT angiography in the evaluation of intracranial aneurysms: image quality, radiation dose, and comparison with 3D rotational digital subtraction angiography. AJR Am J Roentgenol.

[31] Ferda J, Novak M, Mirka H (2009). The assessment of intracranial bleeding with virtual unenhanced imaging by means of dual-energy CT angiography. Eur Radiol.

[32] Cho ES, Chung TS, Oh DK (2012). Cerebral computed tomography angiography using a low tube voltage (80 kVp) and a moderate concentration of iodine contrast material: a quantitative and qualitative comparison with conventional computed tomography angiography. Invest Radiol.

[33] Guggenberger R, Winklhofer S, Osterhoff G (2012). Metallic artefact reduction with monoenergetic dual-energy CT: systematic ex vivo evaluation of posterior spinal fusion implants from various vendors and different spine levels. Eur Radiol.

[34] Shinohara Y, Sakamoto M, Iwata N (2013). Usefulness of monochromatic imaging with metal artifact reduction software for computed tomography angiography after intracranial aneurysm coil embolization. Acta Radiol.

[35] Srinivasan A, Hoeffner E, Ibrahim M, Shah GV, LaMarca F, Mukherji SK (2013). Utility of dual-energy CT virtual keV monochromatic series for the assessment of spinal transpedicular hardware-bone interface. AJR Am J Roentgenol.

[36] Johnson TR (2012). Dual-energy CT: general principles. AJR Am J Roentgenol.

[37] Bolus DN (2013). Dual-energy computed tomographic scanners: principles, comparisons, and contrasts. J Comput Assist Tomogr.

[38] Coursey CA, Nelson RC, Boll DT (2010). Dual-energy multidetector CT: how does it work, what can it tell us, and when can we use it in abdominopelvic imaging?. Radiographics.

[39] Kim SJ, Lim HK, Lee HY (2012). Dual-energy CT in the evaluation of intracerebral hemorrhage of unknown origin: differentiation between tumor bleeding and pure hemorrhage. AJNR Am J Neuroradiol.

[40] Yu L, Leng S, McCollough CH (2012). Dual-energy CT-based monochromatic imaging. AJR Am J Roentgenol.

[41] Grant KL, Flohr TG, Krauss B, Sedlmair M, Thomas C, Schmidt B (2014). Assessment of an advanced image-based technique to calculate virtual monoenergetic computed tomographic images from a dual-energy examination to improve contrast-to-noise ratio in examinations using iodinated contrast media. Invest Radiol.

[42] Lell MM, Ruehm SG, Kramer M (2009). Cranial computed tomography angiography with automated bone subtraction: a feasibility study. Invest Radiol.

[43] Romijn M, Gratama van Andel HA, van Walderveen MA (2008). Diagnostic accuracy of CT angiography with matched mask bone elimination for detection of intracranial aneurysms: comparison with digital subtraction angiography and 3D rotational angiography. AJNR Am J Neuroradiol.

[44] Zhang LJ, Wu SY, Poon CS (2010). Automatic bone removal dual-energy CT angiography for the evaluation of intracranial aneurysms. J Comput Assist Tomogr.

[45] Thomas C, Korn A, Ketelsen D (2010). Automatic lumen segmentation in calcified plaques: dual-energy CT versus standard reconstructions in comparison with digital subtraction angiography. AJR Am J Roentgenol.

[46] Gratama van Andel HA, van Boven LJ, van Walderveen MA (2009). Interobserver variability in the detection of cerebral venous thrombosis using CT venography with matched mask bone elimination. Clin Neurol Neurosurg.

[47] Mokin M, Kan P, Kass-Hout T (2012). Intracerebral hemorrhage secondary to intravenous and endovascular intraarterial revascularization therapies in acute ischemic stroke: an update on risk factors, predictors, and management. Neurosurg Focus.

[48] Fiorelli M, Bastianello S, von Kummer R (1999). Hemorrhagic transformation within 36 hours of a cerebral infarct: relationships with early clinical deterioration and 3-month outcome in the European Cooperative Acute Stroke Study I (ECASS I) cohort. Stroke.

[49] Jang YM, Lee DH, Kim HS (2006). The fate of high-density lesions on the non-contrast CT obtained immediately after intra-arterial thrombolysis in ischemic stroke patients. Korean J Radiol.

[50] Nakano S, Iseda T, Kawano H, Yoneyama T, Ikeda T, Wakisaka S (2001). Parenchymal hyperdensity on computed tomography after intra-arterial reperfusion therapy for acute middle cerebral artery occlusion: incidence and clinical significance. Stroke.

[51] Macdougall NJ, McVerry F, Baird S, Baird T, Teasdale E, Muir KW (2011). Iodinated contrast media and cerebral hemorrhage after intravenous thrombolysis. Stroke.

[52] Kim JT, Heo SH, Cho BH (2011). Hyperdensity on non-contrast CT immediately after intra-arterial revascularization. J Neurol.

[53] Payabvash S, Qureshi M, Khan S (2014). Differentiating intraparenchymal hemorrhage from contrast extravasation on post-procedural noncontrast CT scan in acute ischemic stroke patients undergoing endovascular treatment. Neuroradiology.

[54] • Dinkel J, Khalilzadeh O, Phan CM et al. Technical limitations of dual-energy CT in neuroradiology: 30-month institutional experience and review of literature. J Neurointerv Surg. 2014. doi:10.1136/neurintsurg-2014-011241. *Evaluated the failure modes and limitations of DECT in neuroimaging applications. Ninety percent of DECT analysis was successful, but beam*-*hardening artifacts, the presence of a fourth material or motion can impair DECT*. 10.1136/neurintsurg-2014-011241 24951287

[55] Del Giudice A, D’Amico D, Sobesky J, Wellwood I (2014). Accuracy of the spot sign on computed tomography angiography as a predictor of haematoma enlargement after acute spontaneous intracerebral hemorrhage: a systematic review. Cerebrovasc Dis.

[56] Gazzola S, Aviv RI, Gladstone DJ (2008). Vascular and nonvascular mimics of the CT angiography “spot sign” in patients with secondary intracerebral hemorrhage. Stroke.

[57] Park SY, Kong MH, Kim JH, Kang DS, Song KY, Huh SK (2010). Role of ‘spot sign’ on CT angiography to predict hematoma expansion in spontaneous intracerebral hemorrhage. J Korean Neurosurg Soc.

[58] Hallevi H, Abraham AT, Barreto AD, Grotta JC, Savitz SI (2010). The spot sign in intracerebral hemorrhage: the importance of looking for contrast extravasation. Cerebrovasc Dis.

[59] Tsukabe A, Watanabe Y, Tanaka H (2014). Prevalence and diagnostic performance of computed tomography angiography spot sign for intracerebral hematoma expansion depend on scan timing. Neuroradiology.

[60] Rodriguez-Luna D, Dowlatshahi D, Aviv RI (2014). Venous phase of computed tomography angiography increases spot sign detection, but intracerebral hemorrhage expansion is greater in spot signs detected in arterial phase. Stroke.

[61] Dowlatshahi D, Wasserman JK, Momoli F (2014). Evolution of computed tomography angiography spot sign is consistent with a site of active hemorrhage in acute intracerebral hemorrhage. Stroke.

[62] Chakraborty S, Alhazzaa M, Wasserman JK (2014). Dynamic characterization of the CT angiographic ‘spot sign’. PLoS ONE.

[63] Won SY, Schlunk F, Dinkel J (2013). Imaging of contrast medium extravasation in anticoagulation-associated intracerebral hemorrhage with dual-energy computed tomography. Stroke.

[64] Paul J, Bauer RW, Maentele W, Vogl TJ (2011). Image fusion in dual energy computed tomography for detection of various anatomic structures–effect on contrast enhancement, contrast-to-noise ratio, signal-to-noise ratio and image quality. Eur J Radiol.

[65] Grams AE, Sender J, Moritz R (2014). Dual energy CT myelography after lumbar osteosynthesis. Rofo.

[66] Apfaltrer P, Sudarski S, Schneider D (2014). Value of monoenergetic low-kV dual energy CT datasets for improved image quality of CT pulmonary angiography. Eur J Radiol.

[67] Sudarski S, Apfaltrer P, Nance JW (2013). Optimization of keV-settings in abdominal and lower extremity dual-source dual-energy CT angiography determined with virtual monoenergetic imaging. Eur J Radiol.

[68] Bahner ML, Bengel A, Brix G, Zuna I, Kauczor HU, Delorme S (2005). Improved vascular opacification in cerebral computed tomography angiography with 80 kVp. Invest Radiol.

[69] •• Pomerantz SR, Kamalian S, Zhang D et al. Virtual monochromatic reconstruction of dual-energy unenhanced head CT at 65-75 keV maximizes image quality compared with conventional polychromatic CT. Radiology. 2013;266:318–25. *The authors evaluated monochromatic reconstructions from unenhanced brain DECT to define optimum levels of SNR and CNR in the supratentorial brain and posterior fossa and compared them to conventional CT*. 10.1148/radiol.12111604 23074259

[70] McLellan AM, Daniel S, Corcuera-Solano I, Joshi V, Tanenbaum LN (2014). Optimized imaging of the postoperative spine. Neuroimaging Clin N Am.

[71] Wang Y, Qian B, Li B (2013). Metal artifacts reduction using monochromatic images from spectral CT: evaluation of pedicle screws in patients with scoliosis. Eur J Radiol.

